# Definition of fractal topography to essential understanding of scale-invariance

**DOI:** 10.1038/srep46672

**Published:** 2017-04-24

**Authors:** Yi Jin, Ying Wu, Hui Li, Mengyu Zhao, Jienan Pan

**Affiliations:** 1 School of Resources and Environment, Henan Polytechnic University, Jiaozuo, 454003, China; 2 Collaborative Innovation Center of Coalbed Methane and Shale Gas for Central Plains Economic Region, Henan Province, Jiaozuo, 454003, China

## Abstract

Fractal behavior is scale-invariant and widely characterized by fractal dimension. However, the cor-respondence between them is that fractal behavior uniquely determines a fractal dimension while a fractal dimension can be related to many possible fractal behaviors. Therefore, fractal behavior is independent of the fractal generator and its geometries, spatial pattern, and statistical properties in addition to scale. To mathematically describe fractal behavior, we propose a novel concept of *fractal topography* defined by two scale-invariant parameters, scaling lacunarity (*P*) and scaling coverage (*F*). The scaling lacunarity is defined as the scale ratio between two successive fractal generators, whereas the scaling coverage is defined as the number ratio between them. Consequently, a strictly scale-invariant definition for self-similar fractals can be derived as *D* = log *F* /log *P*. To reflect the direction-dependence of fractal behaviors, we introduce another parameter *H*_*xy*_, a general Hurst exponent, which is analytically expressed by *H*_*xy*_ = log *P*_*x*_/log *P*_*y*_ where *P*_*x*_ and *P*_*y*_ are the scaling lacunarities in the *x* and *y* directions, respectively. Thus, a unified definition of fractal dimension is proposed for arbitrary self-similar and self-affine fractals by averaging the fractal dimensions of all directions in a *d*-dimensional space, which 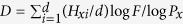. Our definitions provide a theoretical, mechanistic basis for understanding the essentials of the scale-invariant property that reduces the complexity of modeling fractals.

Fractals were originally introduced by Mandelbrot^1^ to describe the fractal behaviors of similar geometries in disordered and irregular objects such as the natural coastlines^1^,^2^,^3^, phenomena in natural and artificial materials^4^,^5^,^6^, porous media^7^,^8^,^9^,^10^, biological structures^11^, rough surfaces^12^,^13^,^14^,^15^, as well as novel application of factuality to complex networks and brain systems^16^,^17^,^18^.

The unique property of fractals is that they are independent of the unit of measurement^3^ and follow a scaling law in the form


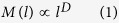


where *M* can be the length of a line or the area of a surface or the volume of an object, and *D* is the fractal dimension. Eq. (1) implies the property of self-similarity, which means that the value of *D* from Eq. (1) remains constant over a range of length scales *l*.

Fractal dimension extends the concept of “dimension”, because it can be a fraction, rather than an integer as in conventional Euclidean space, indicating the degree of complexity of fractal behaviors. Fractal theory now serves as a powerful, perhaps fundamental, tool for characterizing scale-invariance in many fields^19^,^20^,^21^,^22^,^23^,^24^,^25^,^26^,^27^.

In practical applications, *D* can be obtained by a number-size approach or one of its variants, as demonstrated in Eq. (2)


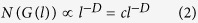


where *N(G(l*)) is the number of similar objects of *G* with characteristic linear dimension *l* and *c* is a constant proportionality. *D* can be determined by the slope of the relationship between log *l* and log*N(G(l*))


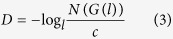


However, the number-size relationship is not a definition, but rather a method for determining the implied fractal dimension. Reexamining fractal theory, *D* is a parameter uniquely determined by the fractal behavior of a similar object (scaling object or fractal generator) in a fractal object, not a parameter that determines such behavior. Different fractal generators following the same fractal behavior will result in the same fractal dimension, while the same fractal generator with different fractal behaviors will lead to different fractal dimensions.

As the variants of the Sierpinski gasket in Fig. 1 show, the fractals in rows 1 and 2 are constructed by different fractal generators, but they share the same fractal dimension, log2/log3, per the number-size relationship (Equation (3)) because their fractal behaviors are the same. The fractal generators for the fractals in rows 2–4 are the same; however, they follow diverse fractal behaviors, resulting in different fractal dimensions (log2/log3, log8/log3, and log5/log6, respectively).

These examples imply that the definition of fractal behavior must satisfy three key requirements: it must be (1) independent of the fractal generator, (2) not constrained by the geometries, spatial patterns, or statistical properties of the fractal generator, and (3) scale-invariant. The number-size relationship does not suffice to preserve fractal behavior information, which hinders the essential understanding of fractal properties and strictly constrains their applications.

## Methods and Discussion

To provide a theoretical, mechanistic basis for understanding the property of scale-invariance, we must mathematically define it per the key requirements we have previously laid out. The first one is **what parameters determine fractal behavior?** For convenience, we call what defines fractal behavior *fractal topography* not only because of the scale and size background, but also that natural structures are often hierarchical, for example, with a sponge-like topology^28^.

To demonstrate fractal topography, it suffices to exhibit structure in a fractal object using a variant of the Sierpinski gasket. As shown in Fig. 2, there are two scale-invariant parameters that determine the fractal behavior of fractal generator *G*: the ratio of the sizes of two successive scaling objects (*l*_*i*_/*l*_*i*+1_) and the ratio of their number (*N(G(l*_*i*+1_))/*N(G(l*_*i*_))).

Fractal topographic information is actually implied in the number-size relationship. For convenience, to mathematically define fractal topography, we first propose two notations:

***Scaling lacunarity (P):*** The unit ratio between two successive fractal generators *G(l*_*i*_) and *G(l*_*i*+1_), with the characteristic dimensions *l*_*i*_ and *l*_*i*+1_ in a fractal object, as


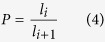


***Scaling coverage (F):*** The numeric ratio between two successive fractal generators *G(l*_*i*_) and *G(l*_*i*+1_) in a fractal object, yields:


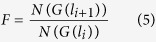


**Figure 1:**
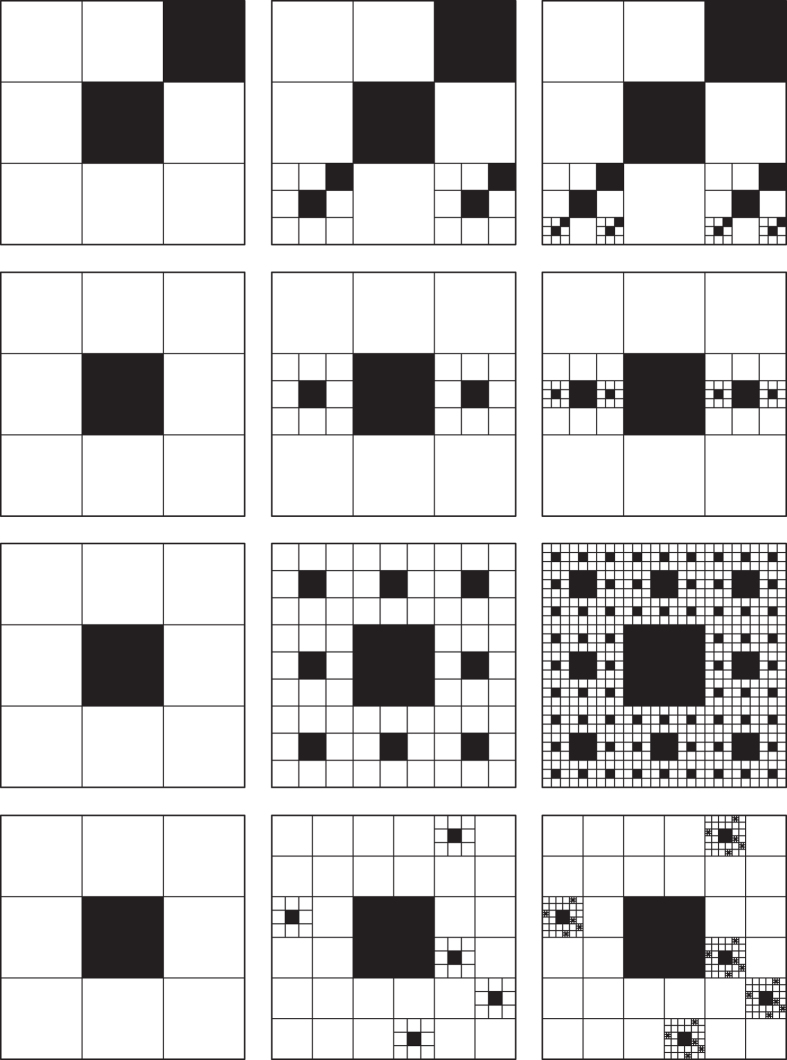
Fractals constructed by different fractal generators with the same fractal behaviors or by the same fractal generators with different fractal behaviors. From left to right in each row, the subfigures demonstrate the construction of fractals with greater detail. Left: the fractal generator is scaled to the characteristic dimension of a fractal object *l*_0_. Center: following a fractal behavior, a simple fractal is constructed. Right: based on the fractal generator and following the fractal behavior, a more complex fractal is obtained in a scale-invariant manner.

**Figure 2:**
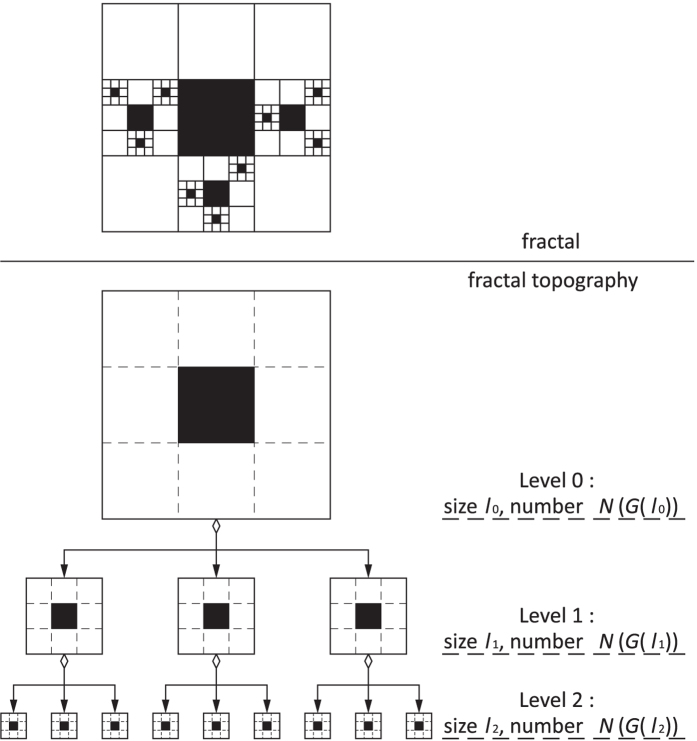
A fractal and its topography for a variant of the Sierpinski gasket.

Apparently, *P* and *F* uniquely determine the fractal behavior of the scaling object in a fractal. These two parameters are dimensionless and scale-invariant, because no matter how we compress or stretch the fractal space, *P* and *F* will not be altered. Taking the properties independent of a fractal generator and its constraints together, demonstrated in Fig. 1, fractal topography is defined by Ω(*P, F*) in this report.

The above discussion answers the question of how to define fractal behavior, but **how does fractal behavior uniquely determine fractal dimension?**

According to Eq. (2), the number of scaling objects of characteristic dimension *l*_*i*_ yields


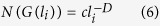


while the number of the successive objects *N(G(l*_*i*_/*P*)) satisfies


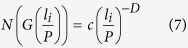


Taking Eqs (4), (5), (6) and (7) into account, we obtain the relationship between the scaling lacunarity and the scaling coverage


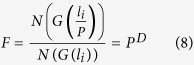


Consequently, a scale-invariant definition of fractal dimension is obtained:


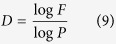


Eq. (9) indicates that the fractal dimension *D* is the exponent of the power-law relationship between *P* and *F*, a dependent parameter determined uniquely by *P* and *F*. Compared with Eq. (3), Eq. (9) preserves fractal topography information and defines fractal dimension in a strictly scale-invariant manner, other than what is implied in the number-size relationship.

**Figure 3:**
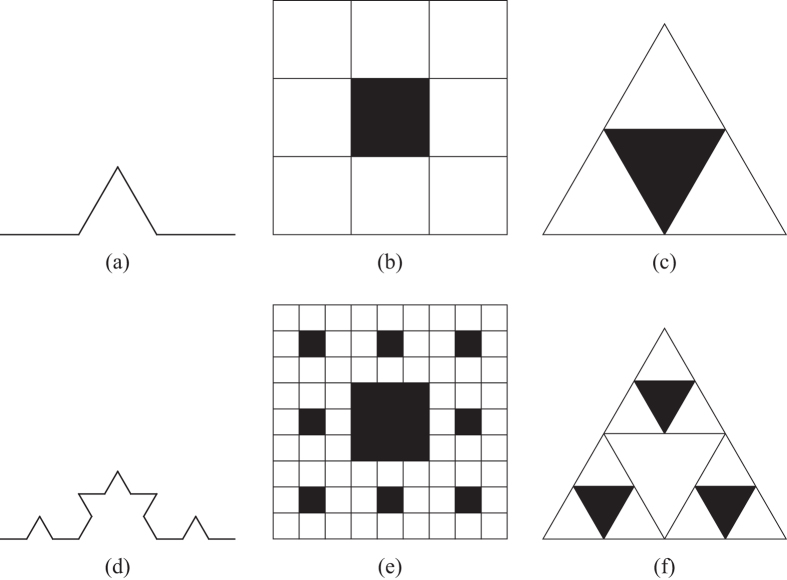
Fractals to demonstrate the validity of Eq. (9). (**a**–**c**) are the initiators of scaling objects of the Koch curve, Sierpinski carpet, and Sierpinski gasket, respectively. For convenience, we denote them as fractal generators. At the next step, each potential subpart is replaced by a reduced replicate of the generator and the fractals are obtained.

To verify Eq. (9), some classic fractals, the Koch curve, Sierpinski carpet, Sierpinski gasket, and Menger Sponge (Fig. 3, here the Menger Sponge not demonstrated), are used to validate it and check its generality.

For a Koch curve, at the stage *n* = 0, *N(G(l*_0_)) = 1 (Fig. 3(a)); at the next stage, *N(G(l*_1_)) = 4 and *l*_1_ = *l*_0_/3 (Fig. 3(b)). Following the definitions of scaling lacunarity and scaling coverage, *P* = *l*_0_/*l*_1_ = 3 and *F* = *N(G(l*_1_))/*N(G(l*_0_)) = 4. Therefore, the fractal dimension of the Koch curve *D* = log4/log3 = 1.2618. The parameters defining and characterizing the fractal topographies of different classic fractals are listed in Table 1. The results are consistent with their theoretical values^3^.

Eq. (9) indicates that:

- *P* and *F* are the intrinsic and basic properties of fractal behavior. *P* controls scaling behavior while *F* determines the degree of space filled by a fractal generator, and together they quantitatively define the topography of a fractal;
- fractal topography uniquely determines fractal dimension, while a fractal dimension can be associated with different fractal topographies/fractal behaviors. For example, the fractal topography with *P* and *F* of *a* and *b* shares the same fractal dimension with those of *a*^*β*^ and *b*^*β*^ for an arbitrary choice of *β*;
- scaling lacunarity and scaling coverage are real scale-invariant dimensionless parameters different from the scale *l* and number *N(G(l*)), and they are also independent of the fractal generator *G(l*_0_) and its geometries, spatial patterns, and statistical properties.

In Fig. 3, the maximum scaling coverages *F*_max_ are 4 (Koch curve), 8 (Sierpinski carpet), and 3 (Sierpinski gasket) at the minimium scaling lacunarities *P*_min_ of 3, 3, and 2, respectively. However, *F* can be fixed to be a value in the series 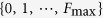, and can even be a fraction in [0, *F*_max_]. For simplicity and without loss of generality, *F* and *P* are set to integers for discussion in this report. Using the Koch curve for explanation, *F* can be assigned to be 0, 1, 2, 3, and 4 while *P* is set to be 3. These fractal dimensions are log0/log3 → −∞^29^, log1/log3, log2/log3, log3/log3 and log4/log3, respectively. Meanwhile, the scaling lacunarity can also be chosen to be larger than *P*_min_. As the fractal in row 4 of Fig. 1, the scaling lacunarity is 6, which is greater than the minimum scaling lacunarity determined by the geometry of the fractal generator.

The definition of the fractal dimension by Eq. (9) is not new and ideas about topography can be found in many previous works^4^,^9^, and can be even tracked back to its original introduction^1^. For convenience of description, we call the fractal topography of Ω{*F*_max_, *P*} the fully-filling scheme, otherwise the partially-filling scheme, where *F*_max_ represents the maximum scaling coverage at a scaling lacunarity of *P*.

In 1967, Mandelbrot defined fractal dimension as 
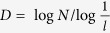
 with a number-size approach, implying the idea of fractal topography. In his demonstration of self-similar curves, 1/(1/4) = *l*_0_/*l*_1_ which is the scaling lacunarity *P*, and *N* = *N(G(l*_1_))/1 = *N(G(l*_1_))/*N(G(l*_0_)) characterizes the scaling coverage. If this were not the case, for example, when *l*_0_ was set to 1/3, 1/2, or any different scale, the calculation results would not be unique; it is only when the scale is “sufficiently fine” that 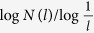 would tend to the limit of *D* and become independent of scale. And the description of scale-invariant phenomena Mandelbrot proposed is very special, because fractal behavior characterized by *D* was heavily dependent on the fractal generators, which means the scaling lacunarity was set to *P*_min_ while the scaling coverage was set to *F*_max_.

In the application of fractal theory in porous media modeling, Perrier and Bird^9^ pointed out the limitations in understanding fractal behavior and proposed a more general filling mode, namely the partially-filling scheme noted before. However, the scaling lacunarity was not broken away from the constraint of fractal generators to be an independent parameter, which means that *P* was fixed to *P*_min_. Turcotte^4^ had proposed a calculation model for *D*, log(*N*_*i*+1_/*N*_*i*_)/log(*l*_*i*_/*l*_*i*+1_), which is exactly the same topographic definition of fractal dimension as Eq. (9). Unfortunately, the physical meanings of the expressions *l*_*i*_/*l*_*i*+1_ and *N*_*i*+1_/*N*_*i*_ were not defined and left the quantitative description of fractal topography elusive, obscuring an essential understanding of scale-invariant properties.

**Figure 4:**
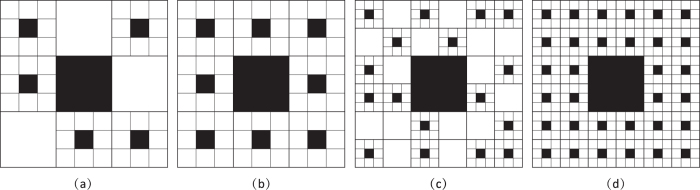
Fractals sharing the same fractal generator but different fractal topographies Ω(*F, P*). The scaling lacunarities of (**a**)–(**d**) are 3, 3, 6, and 6, respectively, while the scaling coverages are 5, 8, 17, and 32. According to Eq. (9), the fractal dimensions of (**a**–**d**) are log5/log3, log8/log3, log17/log6, and log32/log6, respectively.

Based on Ω(*P, F*), together with the fractal generator *G* and its scaling range [*l*_min_, *l*_max_], a self-similar fractal object is uniquely defined as *F*_sim_{Ω(*P, F*), *G*, [*l*_min_, *l*_max_]}, which facilitates the modeling of fractal objects, as Fig. 4 demonstrates.

However, except for the self-similarity, the scale-invariant property might be direction-dependent in a fractal object. Then, **can we unify the definition of fractal dimension for arbitrary fractals?**

Self-affine fractals are objects with scale-invariant and direction-dependent properties^30^. In nature, vertical cross sections are often examples of this type^13^,^31^. A formal definition of a self-affine fractal in a two-dimensional *xy*-space is that *G(ζx, ζ*^*H*^*y*) is statistically similar to *G(x, y*), where *ζ* is a scaling factor and *H* is the Hurst exponent. Based on the scaling lacunarity definition, *G(ζx, ζ*^*H*^*y*) can be written into *G(x/P*_*x*_, *y/P*_*y*_); and by replacing *ζ* by 1/*P*_*x*_, *G(ζx, ζ*^*H*^*y*) takes the form of 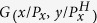. Consequently, we obtain the relationship between *P*_*x*_ and *P*_*y*_ as:


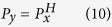


where *P*_*x*_ and *P*_*y*_ are the scaling lacunarities in the *x* and *y* directions respectively. Therefore, the Hurst exponent is scale-invariantly defined by


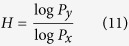


Eq. (11) indicates that the Hurst exponent is a scale-invariant parameter that characterizes the power-law relationship between scaling lacunarities in two different directions. However, the results of Eq. (11) are not constrained in the range of [0, 1]. To clarify the distinction, we call this exponent the general Hurst exponent and express it as *H*_*xy*_ = log*P*_*x*_/log*P*_*y*_.

In a *d*-dimensional space, the fractal dimension in the *i*th direction is denoted by *D*_*i*_ for convenience. According to the additive law^3^,^32^, the fractal dimension *D* is the average of the direction-dependent fractal dimensions, which yields 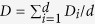. Taking Eq. (9), (11), and the general Hurst exponent together, the fractal dimension of a self-affine fractal is expressed by


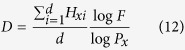


To be consistent with the value range of the Hurst exponent, [0, 1], we denote the maximum scaling lacunarity in all directions of a fractal object by *P*_max_. According to Eq. (11), the Hurst exponent *H*_*i*_ in the *i*th direction yields log*P*_*i*_/log*P*_max_. Therefore, the general definition of arbitrary fractals is then rearranged into


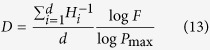


If all *P*_*i*_ are the same, *H*_*i*_ = 1 are all satisfied. Thus, Eq. (13) is same as Eq. (9), which characterizes self-similar fractal behaviors. Otherwise, it depicts the direction-dependent fractal behavior of self-affine fractal objects. Obviously, Eq. (9) is only a special case of the general definition of fractal dimension (Equation (13)) to characterize fractal behaviors.

## Conclusion

Based on the theoretical, mechanistic basis for understanding the nature of fractal behaviors, two parameters are proposed to define the fractal topography that uniquely determine fractal behavior and dimension. These two parameters, scaling lacunarity and scaling coverage, are independent of the fractal generator and the scale and they are intrinsic properties of a fractal topography. However, owing to anisotropic origins, fractal topography may appear direction-dependent, meaning that the scaling lacunarities are different in different directions. In this study, we find that the physical meaning of the Hurst exponent is a scale-invariant exponent that characterizes the power-law relationship of scaling lacunarities in two different directions. Consequently, a unified definition of fractal dimension for arbitrary fractals is proposed by averaging the fractal dimensions of all directions in a strictly scale-invariant manner.

**Figure 5:**
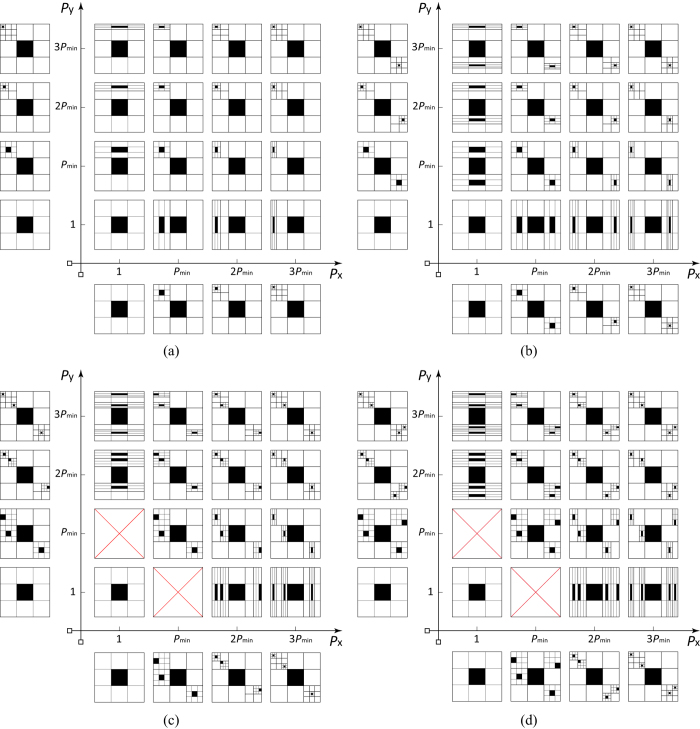
Construction of self-same, self-similar, and self-affine objects following different fractal topographies. All the fractals generated from the same generator but with different scaling lacunarities *P* and scaling coverages *F*. When *P*_*x*_ = *P*_*y*_ = 1, the generated objects are self-same; while *P*_*x*_ = *P*_*y*_ ≠ 1, the generated objects are self-similar; else the generated objects are self-affines. The scaling coverages *F* in (**a**)–(**d**) are 1–4, respectively. In each subfigure, as *P*_*x*_/*P*_*y*_ deviates further from 1, the anisotropy of the fractal increases.

Apparently, Eq. (13) unifies the definition of fractal dimension for arbitrary fractals, including self-samenesses, self-similarites and self-affinities, due to the proposal of fractal topography. In addition to that, fractal topography provides an essential understanding of fractal behavior that eases the implementation and reduces the modeling complexity of disordered and irregular fractal objects, as demonstrated in some cases of two-dimensional porous media in Fig. 5. Although our definitions are derived in view of regular geometries, their practical application is straightforward in a statistical form.

## Additional Information

**How to cite this article**: Jin, Y. *et al*. Definition of fractal topography to essential understanding of scale-invariance. *Sci. Rep.* **7**, 46672; doi: 10.1038/srep46672 (2017).

**Publisher's note:** Springer Nature remains neutral with regard to jurisdictional claims in published maps and institutional affiliations.

## Figures and Tables

**Table 1:** The fractal topography information of classic fractals and their fractal dimensions calculated by Eq. (9).

Fractals	*P*	*F*	*D*
Koch curve	3	4	1.2618
Sierpinski carpet	3	8	1.8927
Sierpinski gasket	2	3	1.5842
Menger Sponge	3	26	2.9656

## References

[b1] Mandelbrot B. B. How long is the coast of britain? statistical self-similarity and fractional dimension. Science 156, 636–8 (1967). 17837158 10.1126/science.156.3775.636

[b2] Mandelbrot B. B. Stochastic models for the earth’s relief, the shape and the fractal dimension of the coastlines, and the number-area rule for islands. Proc. Natl. Acad. Sci. 72, 3825–8 (1975). 16578734 10.1073/pnas.72.10.3825 PMC433088

[b3] Mandelbrot B. B. The fractal geometry of nature (Macmillan, New York, 1983).

[b4] Turcotte D. L. Fractals and Choas in Geology and Geophysics (Cambridge University Press, New York, 1997).

[b5] Bejan A. & Lorente S. Constructal theory of generation of configuration in nature and engineering. J. Appl. Phys. 100, 041301 (2006).

[b6] Cheng Q. M. Singularity theory and methods for mapping geochemical anomalies caused by buried sources and for predicting undiscovered mineral deposits in covered areas. J. Geochem. Explor. 122, 55–70 (2012).

[b7] Krohn C. E. & Thompson A. H. Fractal sandstone pores: Automated measurements using scanningelectron- microscope images. Phys. Rev. B. 33, 06366 (1986). 10.1103/physrevb.33.6366 9939189

[b8] Smidt J. M. & Monro D. M. Fractal modeling applied to reservoir characterization and flow simulation. Fractals 6, 401–408 (1998).

[b9] Perrier E., Bird N. & Rieu M. Generalizing the fractal model of soil structure: the pore-solid fractal approach. Geoderma 88, 137–164 (1999).

[b10] Jin Y., Zhu Y. B., Li X., Zheng J. L. & Dong J. B. Scaling invariant effects on the permeability of fractal porous media. Transport Porous Med. 109, 433–453 (2015).

[b11] West G. B., Brown J. H. & Enquist B. J. A general model for the origin of allometric scaling laws in biology. Science 276, 122–6 (1997). 9082983 10.1126/science.276.5309.122

[b12] Brown S. R. & Scholz C. H. Broad bandwidth study of the topography of natural rock surfaces. J. Geophys. Res. 90, 12512–12575 (1985).

[b13] Dubuc B., Quiniou J. F., Roquescarmes C., Tricot C. & Zucker S. W. Evaluating the fractal dimension of profiles. Phys. Rev. A 39, 1500–1512 (1989). 10.1103/physreva.39.1500 9901387

[b14] Buzio R., Boragno C., Biscarini F., De Mongeot F. B. & Valbusa U. The contact mechanics of fractal surfaces. Nat. Mater. 2, 233–236 (2003). 12690395 10.1038/nmat855

[b15] Jin Y., Dong J. B., Zhang X. Y., Li X. & Wu Y. Scale and size effects on fluid flow through self-affine rough fractures. Int. J. Heat Mass Tran. 105, 443–451 (2017).

[b16] Song C., Makse H. A. & Gallos L. K. Scaling of degree correlations and its influence on diffusion in scale-free networks. Phys. Rev. Lett. 100, 248701 (2008). 18643633 10.1103/PhysRevLett.100.248701

[b17] Galvao V. . Modularity map of the network of human cell differentiation. Proceedings of the National Academy of Sciences 107, 5750–5755 (2010). 10.1073/pnas.0914748107 PMC2851936 20220102

[b18] Gallos L., Sigman M. & Makse H. The conundrum of functional brain networks: Small-world efficiency or fractal modularity. Frontiers in Physiology 3, 123 (2012). 22586406 10.3389/fphys.2012.00123 PMC3345943

[b19] Ma D., Stoica A. D. & Wang X. L. Power-law scaling and fractal nature of medium-range order in metallic glasses. Nat. Mater. 8, 30–34 (2009). 19060888 10.1038/nmat2340

[b20] Jin Y., Song H. B., Hu B., Zhu Y. B. & Zheng J. L. Lattice boltzmann simulation of fluid flow through coal reservoir’s fractal pore structure. Sci. China Earth Sci. 56, 1519–1530 (2013).

[b21] Wang B. Y. . Derivation of permeability-pore relationship for fractal porous reservoirs using series-parallel flow resistance model and lattice boltzmann method. Fractals 22, 1440005 (2014).

[b22] Dingal P. C. D. P. . Fractal heterogeneity in minimal matrix models of scars modulates stiff-niche stem-cell responses via nuclear exit of a mechanorepressor. Nat. Mater. 14, 951–60 (2015). 26168347 10.1038/nmat4350 PMC4545733

[b23] Zheng X. . Multiscale metallic metamaterials. Nat. Mater. 15, 1100–1106 (2016). 27429209 10.1038/nmat4694

[b24] Ding D., Zhao Y., Feng H., Si B. & Hill R. L. A user-friendly modified pore-solid fractal model. Sci. Rep. 6, 39029 (2016). 27996013 10.1038/srep39029 PMC5171870

[b25] Namazi H. & Kulish V. V. Fractal based analysis of the influence of odorants on heart activity. Sci. Rep. 6, 38555 (2016). 27929045 10.1038/srep38555 PMC5144066

[b26] Cheng Q. Fractal density and singularity analysis of heat flow over ocean ridges. Sci. Rep. 6, 19167 (2016). 26757680 10.1038/srep19167 PMC4725826

[b27] Jin Y., Li X., Zhao M., Liu X. & Li H. A mathematical model of fluid flow in tight porous media based on fractal assumptions. Int. J. Heat Mass Tran. 108, Part A, 1078–1088 (2017).

[b28] Carpinteri A. & Pugno N. Are scaling laws on strength of solids related to mechanics or to geometry? Nat. Mater. 4, 421–423 (2005). 15928689 10.1038/nmat1408

[b29] Ghanbarian-Alavijeh B. & Hunt A. G. Comments on “more general capillary pressure and relative permeability models from fractal geometry” by kewen li. J. Contam. Hydrol. 140–141, 21–23 (2012). 10.1016/j.jconhyd.2012.08.004 22982613

[b30] Mandelbrot B. B. Self-affine fractals and fractal dimension. Phys. Scripta 32, 257–260 (1985).

[b31] Dubuc B., Zucker S. W., Tricot C., Quiniou J. F. & Wehbi D. Evaluating the fractal dimension of surfaces. Proc. R. Soc. London, Ser. A 425, 113–127 (1989).

[b32] Sreenivasan K. R. Fractals and multifractals in fluid turbulence. Annu. Rev. Fluid Mech. 23, 539–604 (1991).

